# Torsional resistance of three ProTaper rotary systems

**DOI:** 10.1186/s12903-019-0820-7

**Published:** 2019-06-21

**Authors:** Abdullah Alqedairi, Hussam Alfawaz, Bader Abualjadayel, Mohammad Alanazi, Ahmad Alkhalifah, Ahmed Jamleh

**Affiliations:** 1 Department of Restorative Dental Sciences, College of Dentistry, King Saud University, Riyadh, Kingdom of Saudi Arabia; 2 King Abdullah International Medical Research Centre, Restorative and Prosthetic Dental Sciences, College of Dentistry, Ministry of National Guard Health Affairs, King Saud bin Abdulaziz University for Health Sciences, P.O. Box 22490, Riyadh, 11426 Saudi Arabia

**Keywords:** ProTaper gold, ProTaper next, ProTaper universal, Scanning electron microscope, Torsional resistance

## Abstract

**Background:**

The aim of this study is to compare the torsional resistance of the available ProTaper rotary systems, namely, ProTaper Universal (PTU), ProTaper Next (PTN), and ProTaper Gold (PTG).

**Methods:**

A total of 195 files from the three systems distributed into 13 groups (PTU-S1, PTU-S2, PTU-F1, PTU-F2, PTU-F3, PTG-S1, PTG-S2, PTG-F1, PTG-F2, PTG-F3, PTN-X1, PTN-X2 and PTN-X3) were subjected to torsional fatigue until failure. The torsional test was performed according to ISO 3630-1, where each file was placed in a straight position to eliminate the influence of cyclic fatigue. The Kruskal–Wallis test was conducted to compare the mean maximum torques and angular deflections at fracture for the groups, and the Mann–Whitney test was performed for pairwise comparisons. The significance level was set at 0.05 and the fractured surfaces were examined under a scanning electron microscope.

**Result:**

Among the tested files, PTG-S1 had the lowest torsional fatigue resistance, whereas PTU-F2 and PTU-F3 had the highest torsional resistance. The scanning electron microscope showed typical features of torsional failure.

**Conclusion:**

The new ProTaper systems (PTG and PTN) did not show improved torsional resistance in comparison with PTU.

## Background

Currently, the mechanical preparation of the root canal system can be performed safely with the use of nickel titanium (NiTi) rotary files. This rotary file’s flexibility showed a huge improvement in endodontic treatment, wherein it can prepare the root canal quickly with less iatrogenic errors while maintaining the original canal anatomy [1, 2]. Despite these benefits, the NiTi file can fracture unexpectedly, which is a complication that adversely affects the prognosis [3]. Any preparation stress induced on the file can result in file strain, which leads to fatiguing and eventually file fracture, especially when the stress exceeds the strength of the NiTi file. File fatigue is a well-known mechanism for file fracture caused by cyclic fatigue, torsional failure, or both [4]. Cyclic fatigue occurs while the file rotates in a curved geometry, wherein the stress is at its highest at the area of maximum curvature, producing alternating compression and tension cycles until fracture [5, 6]. Torsional fatigue failure occurs when the torque resulting from contact between the file and canal wall exceeds the torsional strength of the file, or by twisting the file through its longitudinal axis at one end while the tip or another part of the file is locked in the canal [1, 4, 7]. The lifetime of NiTi files may be influenced by many factors, such as file geometry, metal surface treatment, thermal treatment, and metallurgic characterization of the NiTi alloys [8–11].

Increasing the resistance to file fracture has been the main goal of manufacturers in developing new file systems to improve the safety and effectiveness of canal preparation through innovative design and manufacturing processes [12–14]. Modification in the manufacturing process or the use of new alloys, changing the taper over the length of the cutting blades, and adjusting the instrument’s cross-sectional design are methods used to enhance the file’s clinical performance [12, 15, 16].

Currently, three ProTaper rotary systems (Dentsply Sirona) are available in the market, namely, ProTaper Universal (PTU), ProTaper Next (PTN), and ProTaper Gold (PTG). PTU is manufactured from super-elastic conventional NiTi alloy. It has shaping (S1 (size 17, .02 taper) and S2 (size 20, .04 taper)) and finishing (F1 (size 20, .07 taper), F2 (size 25, .08 taper), and F3 (size 30, .09 taper)) files. PTN is manufactured from martensitic (M-Wire) NiTi alloy subjected to thermo-mechanical processing. It has X1 (size 17, .04 taper), X2 (size 25, .06 taper), and X3 (size 30, .07 taper) files. The design of these files is different from that of the PTU files. The PTN files include variable tapers and an off-centered rectangular cross-section. The martensitic wire technology, in combination with the unique design, was shown to enhance flexibility and fracture resistance [17, 18]. The action of X1 can replace PTU-S1, PTU-S2, and PTU-F1 files [19]. Recently, ProTaper Gold (PTG) has been introduced and it has the same design, geometry, and features as those of the PTU files. However, it has been developed with proprietary advanced metallurgy from Gold-wire NiTi, which makes it more flexible than PTU [18, 20, 21].

Torsional resistance is one of the most significant mechanical properties of the NiTi alloy, which can affect the clinical performance of endodontic files, especially in narrow canals. Previous studies have tested the torsional resistance of specific sizes of the ProTaper systems [16, 17, 20, 22–27]. However, investigating the torsional behavior of the commonly used series of files of the available ProTaper systems has not been addressed adequately. Thus, the present study was conducted to compare the torsional fatigue resistance of the ProTaper files of different systems, namely, the conventional wire, M-Wire, and Gold-wire NiTi. The null hypothesis was that there is no difference in the torsional resistance among the tested ProTaper files.

## Methods

A total of 195 new files distributed into 13 groups (PTU-S1, PTU-S2, PTU-F1, PTU-F2, PTU-F3, PTN-X1, PTN-X2, PTN-X3, PTG-S1, PTG-S2, PTG-F1, PTG-F2, and PTG-F3) (*n* = 15) were used for torsional evaluation.

The torsional test was conducted according to ISO 3630-1 (International Organization for Standardization, 1992) [28] by using a torsion tester (WP 500 torsion tester-30 Nm, Gunt Hamburg, Germany). A portion of 3 mm from the file tip was clamped with a pin vise. The file was rotated clockwise as viewed from the shank end and the test speed was set to 2 rpm. The device was calibrated before the test. The torques at failure and maximum angular deflections were recorded.

Fractured files with the highest and lowest torque values from each group, totaling 26 files, were selected for fractographic analysis by using a scanning electron microscope (SEM; JEOL 6360LV Scanning Electron Microscope, Japan). Each file was cleaned in absolute alcohol and fixed on a metallic stub to evaluate the fracture area at 200X, 500X, and 1000X magnification levels.

An additional new specimen of each file type (13 in total) was sectioned at 3 mm from the tip (D3) to observe and measure the cross-sectional surface area under a stereomicroscope.

### Statistical analysis

Because the torque distributions at failure and angular deflection were found to be abnormal by the Shapiro–Wilk test (*P* = 0.000), the Kruskal–Wallis and Mann–Whitney tests were conducted to determine the statistical significance among the tested groups. The significance was determined at a 5% level.

## Results

The results of the torsional tests are summarized in Table 1, which present the mean (± standard deviation), median, and range values of both torque at failure and angular deflection at failure of the tested file types.

**Table 1:** Descriptive data of the torque at failure and angular deflection in the tested systems

		Surface area at D3 (mm^2^)	Torque at failure (gcm)			Angular deflection (degree)		
			Mean ± SD	Median	Range	Mean ± SD	Median	Range
PTU	S1	0.022	68.8 ± 25.1	77.6^af^	25.5–101.2	347.9 ± 37.6	355^aeg^	270–420
	S2	0.024	112.5 ± 30.5	108.6^b^	60.6–182.2	367 ± 48.5	365^af^	290–495
	F1	0.048	100.6 ± 36.4	93.9^abg^	63.3–169.8	380.3 ± 48.1	375^bf^	295–480
	F2	0.121	141.7 ± 37.6	136.3^c^	90.82–235.5	346.7 ± 56.6	350^aeg^	275–470
	F3	0.149	196.2 ± 20.9	189.8^d^	168.4–250.8	559 ± 62.4	540^c^	450–690
PTG	S1	0.022	43.4 ± 14.9	41.6^e^	22.2–80	361.7 ± 53.1	370^abdef^	280–440
	S2	0.024	73.7 ± 33.3	66.3^f^	37.4–179.0	331.0 ± 39.9	335^e^	260–385
	F1	0.048	94 ± 23.1	91.4^b^	37.8–129.4	393.2 ± 60.6	390^f^	285–495
	F2	0.121	117.4 ± 32	115.5^bcg^	70.6–201.2	371.3 ± 50.4	370^fg^	280–495
	F3	0.149	166.4 ± 17.2	167.4^h^	135.1–194.5	580.3 ± 61.7	580^c^	480–670
PTN	X1	0.048	59.9 ± 13.5	58.8^f^	36.7–82.2	291.6 ± 35.8	280^h^	220–360
	X2	.094	95.8 ± 38.9	92a^b^	44.3–185.1	303.1 ± 32.5	310^h^	245–355
	X3	0.137	115.4 ± 17.6	116.7^i^	81.4–142.9	303.7 ± 26.3	300^h^	270–355

Different superscripts indicate statistical significance

Amongst all the tested files, PTG-S1 and PTU-F3 demonstrated the lowest and highest torque at failure, respectively (*P* < 0.05). There was a tendency of torque at failure increasing as the file cross-sectional area increased (Table 1). Within each system, there were significant differences between all the file types, except between PTU-S1 and PTU-F1 and PTU-S2 and PTU-F1, and between PTG-F1 and PTG-F2, which were found to be comparable.

The comparison of the PTU and PTG shaping files revealed that the PTU-S1 and PTU-S2 files had significantly higher torsional failure resistance than the PTG-S1 and PTG-S2 files, respectively (*P* < 0.01). In the comparison of the PTU-F1, PTG-F1, and PTN-X1 files, the PTN-X1 was found to have significantly the lowest torsional resistance (*P* < 0.001), whereas the PTU-F1 and PTG-F1 files had comparable values (*P* = 0.74). The comparison of the PTU-F2, PTG-F2, and PTN-X2 files revealed that the PTN-X2 file had a significantly lower torsional resistance than the PTU-F2 file (*P* < 0.001). In the comparison of the PTU-F3, PTG-F3, and PTN-X3 files, the PTN-X3 was found to have significantly the lowest torsional resistance, followed by the PTG-F3 and PTU-F3 files (P < 0.001).

When a similar analysis was performed for angular deflection at fracture (Table 1), it was found that the PTU-F3 and PTG-F3 files were comparable and had significantly the highest angular deflection values (*P* < 0.05). The three PTN files showed comparable results and had significantly the lowest angular deflections in comparison with the other file types (*P* < 0.05).

**Fig. 1:**
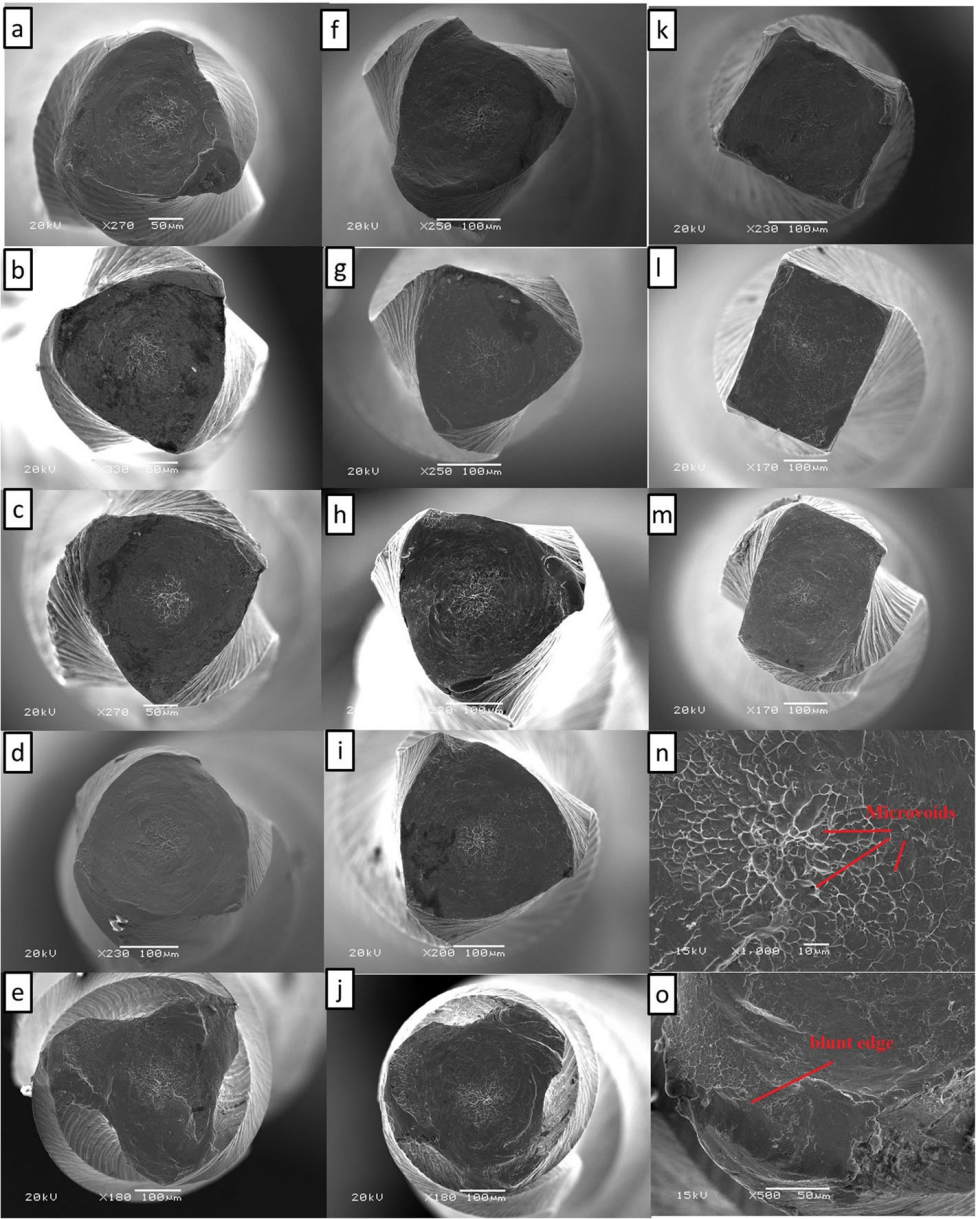
Scanning electron microscopic images of ProTaper Universal, ProTaper Gold, and ProTaper Next instruments after the torsional resistance testing revealing circular abrasion streaks and skewed dimples in the center of the fractured surfaces. **a** PTU-S1, **b** PTU-S2, **c** PTU-F1, **d** PTU-F2, **e** PTU-F3, **f** PTG-S1, **g** PTG-S2, **h** PTG-F1, **i** PTG-F2, **j** PTG-F3, **k** PTN-X1, **l** PTN-X2, and **m** PTN-X3. **n** and **o** show the fracture characteristics that occurred after the torsional resistance testing

Under the SEM, all the instruments exhibited similar torsional fatigue behavior. The fractured cross-sectional surfaces revealed typical features of torsional failure, including concentric abrasion pattern, and a dimpled surface with micro-voids in the middle (Fig. 1).

At D3, the surface areas of S1, S2, F1, F2, and F3 in PTU and PTG were 0.022, 0.024, 0.048, 0.121, and 0.149 mm^2^, respectively, whereas the PTN-X1, PTN-X2, and PTN-X3 had surface areas of 0.48, 0.094, and 0.137 mm^2^, respectively (Table 1).

## Discussion

Differences among the torsional resistance data of the tested files were detected during evaluation. PTG-S1 and PTU-F3 showed the lowest and highest torque at failure values, respectively (Table 1). Furthermore, the lowest angular deflections were detected with the three PTN files. Therefore, the null hypothesis was rejected.

Torsional strength indicates the file ability to get twisted before fracture occurs. This property was shown to be influenced by many factors, such as file size, design, alloy’s chemical composition, and manufacturing processes [29–31]. Within each group, it was found that the maximum torque increased as the file size increased. Furthermore, the tested PTN files (X1, X2, and X3) revealed lower torsional resistances than their respective PTU and PTG files (F1, F2, and F3). These findings could be attributed to the file design and cross-sectional area, where PTN has a rectangular cross section whereas the PTU and PTG have convex triangular cross-sections. These observations are consistent with those of previous studies, wherein an increase in the central core diameter of the files was reported to enhance its resistance to the torsional stress [16, 17, 22, 23, 25, 27, 32]. However, another study found that the PTN-X2 had the highest torsional resistance, followed by PTU-F2 and PTG-F2 [24]. This conflict may be due to the study design, where they used a different device that was not compatible with the ISO 3630-1 specification. Besides that, they claimed that the off-centered cross-sectional design could be the main reason for the improved torsional behavior. However, the in vitro torsional testing performed does not provide a suitable condition to test the effect of file geometry. Rather, it provides file behavior at the area where it is held. In this study, the surface areas of the files were measured in the area subjected to torsional fatigue and it was found that the PTN files have smaller surface areas than their corresponding file types in PTU and PTG. Moreover, alterations in file taper might explain the differences in torsional behavior.

The manufacturing process, alloy properties and thermal treatment could influence the fatigue resistance behaviors of rotary files [18]. Thermal treatment of NiTi alloy is considered one of the most effective methods to enhance and modify the mechanical properties of the alloy [20]. The present results showed that PTU files had higher torsional resistance than their respective PTG files except F1 and F2 files. Similarly, Elnaghy and Elsaka [20] reported that the PTG-F2 file did not show improved resistance to torsional stress in comparison with the PTU-F2 file. However, Kaval et al. [26] found that the PTG-F2 files had a higher torsional resistance than the PTU-F2 files. Although alterations in the manufacturing process enhanced the cyclic fatigue resistance of PTG [20, 26, 33] and made its files more flexible than the PTU files [18, 20, 21], it did not improve the torsional behavior. This may be attributed to manufacturing process of the PTG files to keep them at martensitic phase at body temperature with advanced metallurgy and special thermal treatment [18]. This allows to have a greater amount of deformation than the conventional NiTi alloy [16].

Although higher angular deflection of the file before the fracture could be beneficial to prevent intracanal fracture, it may have no clinical significance because one complete rotation will occur in 0.2 s at a speed of 300 rpm [34]. Moreover, in agreement with the previous studies, the angular deflection measurements did not correlate with their corresponding torque at failure values [35].

It is noteworthy that the current study subjected 3 ProTaper systems to the same experimental setting. Although the present methodology does not mimic the clinical setting, the findings could give information about which file system can be used based on the canal geometry. Evaluating the mechanical properties of the NiTi files and their performance is essential for clinicians to select the system that improves clinical outcomes [14]. Once the torsional strength of the file is exceeded, intracanal file fracture can occur [4]. Clinically, the fracture can be caused due to the frictional forces generated between the file and root canal dentin, whereby preparation of narrow and constricted canals can subject rotary NiTi instruments to high torsional loads, mainly with small instruments and in the apical third of the canals [36]. In such conditions, the conventional PTU files with their high torsional resistance are suggested for use to avoid the intracanal file fracture [27, 37].

## Conclusions

This study evaluated the torsional resistance of different ProTaper systems that are currently used by clinicians. Differences were detected among the torsional resistance data of the tested files. The results showed that the PTG-S1 and PTU-F3 had the lowest and highest torque at failure values, respectively. The lowest angular deflections were detected with the three PTN files. Within the limitations of this study, the new ProTaper systems did not show higher torque at failure data in comparison with PTU.

## Data Availability

The datasets used and/or analysed during the current study are available from the corresponding author on reasonable request.

## Abbreviations

NiTi: nickel titanium
PTG: ProTaper Gold
PTN: ProTaper Next
PTU: ProTaper Universal
SEM: scanning electron microscope
